# Modification and co-option of leaf developmental programs for the acquisition of flat structures in monocots: unifacial leaves in Juncus and cladodes in Asparagus

**DOI:** 10.3389/fpls.2013.00248

**Published:** 2013-07-09

**Authors:** Hokuto Nakayama, Takahiro Yamaguchi, Hirokazu Tsukaya

**Affiliations:** ^1^Department of Bioresource and Environmental Sciences, Faculty of Life Sciences, Kyoto Sangyo UniversityKyoto, Japan; ^2^Department of Biological Sciences, Graduate School of Science, The University of TokyoTokyo, Japan

**Keywords:** *Asparagus*, cladode, evolution, gene regulatory networks, *Juncus*, leaf, leaf-like organ, unifacial leaf

## Abstract

It has been suggested that modification and co-option of existing gene regulatory networks (GRNs) play an important role in the morphological diversity. In plants, leaf development is one of active research areas, and the basic GRN for leaf development is beginning to be understood. Moreover, leaves show wide variation in their form, and some of this variation is thought to be the result of adaptation. Thus, leaves and leaf-like organs are an emerging and interesting model to reveal how existing GRNs give rise to novel forms and architectures during evolution. In this review, we highlight recent findings in evo-devo studies, especially on *Juncus* unifacial leaves, which are composed of lamina with abaxialized identities, and *Asparagus* cladodes, which are leaf-like organs at the axils of scale leaves. Based on these studies, we discuss how flat structures have evolved and morphologically diversified in shoot systems of monocot species, focusing on the modification and co-option of GRN for leaf development.

## INTRODUCTION

Plants display remarkable morphological diversities (Bell, 2008), which are largely a result of morphological variations of the shoot consisting of leaves, internodes, and axillary buds (flowers are considered as reproductive shoots consisted of modified leaves; von Goethe, 1790; Tsukaya, 1995; Honma and Goto, 2001). Recent studies have revealed the mechanisms underlying the morphological diversity of shoots (Garcês et al., 2007; Kimura et al., 2007). Importantly, many of these studies suggested that the modification and co-option of existing developmental gene regulatory networks (GRNs) play an important role in the development of novel forms and organs. For example, a study of MADS-box genes, which are transcription factors that specify floral organ identities, indicated that modification of GRN for floral organ identity has led to acquisition of unusual floral organs (Kramer et al., 2007). Likewise, studies of class I *KNOTTED-LIKE HOMEOBOX* (*KNOX1*) genes, which are required for initiation and maintenance of the shoot apical meristem (SAM), indicated that co-option and modification of GRN for the meristem development play a role in the morphological diversity of leaves (Hay and Tsiantis, 2006; Piazza et al., 2010). GRNs are considered connected components, which can be rewired and transplanted into new developmental contexts. Thus, elucidating how existing GRNs give rise to novel forms and architectures is important for understanding both morphological diversity and adaptation processes.

Here, we discuss the modification and co-option of GRN for the establishment of adaxial–abaxial (ad–ab) polarity, using two examples: *Juncus* unifacial leaves, in which leaf blades have only the abaxial identity, and *Asparagus* cladodes, which are leaf-like organs in the axils (Yamaguchi et al., 2010; Nakayama et al., 2012a). Based on these results, we discuss the importance of modification and co-option of GRN for the acquisition of flat structures in monocots.

## BASIC GRNs OF LEAF ORGANOGENESIS REGARDING LEAF INITIATION AND ESTABLISHMENT OF DORSIVENTRALITY

The mechanisms underlying the morphological diversity of leaves remain poorly understood, whereas the basic mechanisms of leaf development are beginning to be characterized (Tsukaya, 2006; Koenig and Sinha, 2010; Moon and Hake, 2010; Horiguchi and Tsukaya, 2011; Tsukaya, 2013). Since leaf primordia develop from a group of undifferentiated cells on the flank of the SAM, initiation of leaf primordia requires a transition of cell fate from pluripotent to determinate. In *Arabidopsis*, *KNOX1* genes are known to be the transcription factors that maintain meristem activity in shoots (Long et al., 1996). Therefore, the expression of *KNOX1* genes is down-regulated at initiation sites of leaf primordia within the SAM (Long et al., 1996; Guo et al., 2008). This repression is partially mediated by a protein complex containing ASYMMETRIC LEAVES 1 (AS1), a MYB domain transcription factor, and ASYMMETRIC LEAVES 2 (AS2), a LOB domain protein (Xu et al., 2003; Ueno et al., 2007), and this interaction is known as the *A**SYMMETRIC LEAVES1*/*R**OUGHSHEATH2*/*P**HANTASTICA-KNOX* (*ARP*-*KN-OX*) module.

After the initiation of leaf primordia marked by the repression of *KNOX1*, an ad–ab polarity is established within leaf primordia. Cell fates in the root meristem are determined mainly by a positional cue rather than cell lineage (van den Berg et al., 1995; Scheres, 2001), and the positional cue plays an important role in the establishment of ad–ab polarity in leaf primordia (Wardlaw, 1949; Steeves and Sussex, 1989). Leaf primordia innately possess positional information against the SAM: the adaxial side is derived from cells adjacent to the SAM, while the abaxial side is derived from more distant cells (Kidner and Timmermans, 2010). Microsurgical experiments have demonstrated that communication between the SAM and leaf primordia is required for the establishment of ad–ab polarity, and suggested that the SAM may release signal molecules (Sussex, 1951; Reinhardt et al., 2005). Recently, it has been demonstrated that succinic semialdehyde or similar derivatives may be SAM signal molecules (Toyokura et al., 2011). Moreover, many studies using various model species have demonstrated that ad–ab polarity is established via mutually antagonistic interactions between adaxial and abaxial determinants (reviewed in Chitwood et al., 2007; Yamaguchi et al., 2012). For example, AS1 represses the abaxial determinant, *ETTIN*/*AUXIN* *RESPONSE* *FACTOR3* (*ETT*/*ARF3*), as a complex with AS2 (Iwakawa et al., 2007). Moreover, type III *HOMEODOMAIN-LEUCINE ZIPPER* (*HD-ZIP III*) transcription factors, *PHABULOSA* (*PHB*), *REVOLUTA* (*REV*), and *PHAVOLUTA *(*PHV*), specify adaxial fate in leaf primordia (McConnell and Barton, 1998; McConnell et al., 2001; Emery et al., 2003). Meanwhile, transcription factors such as *ARF3*, *ARF4*, *YABBY*, and *KANADI* (*KAN*) specify abaxial fate (Eshed et al., 2001; Kerstetter et al., 2001; Pekker et al., 2005; Sarojam et al., 2010). Additionally, two small RNAs play an important role in establishment of ad–ab polarity. miR166 cleaves mRNA of *HD-ZIP III* on the abaxial side of leaf primordia (Rhoades et al., 2002; Emery et al., 2003). On the other hand, tasiR-ARF, which is derived from non-coding TAS3 precursor transcripts, represses the expression of *ARF3* and *ARF4* on the adaxial side (Fahlgren et al., 2006; Hunter et al., 2006). Therefore, miR165/166 and tasiR-ARF also specify the abaxial and adaxial cell fate, respectively. According to the established ad–ab polarity, the leaf lamina expands along the medio-lateral axis because outgrowth of the leaf lamina is promoted at the juxtaposition between the adaxial and abaxial sides of leaf primordia (Waites and Hudson, 1995). During outgrowth, it is known that the establishment of the middle domain marked by expression of *WUSCHEL-RELATED HOMEOBOX* (*WOX*) genes is required (Nakata et al., 2012). *WOX1* and *PRESSED FLOWER* (*PRS*), which are negatively regulated by *KAN* and positively regulated by *FILAMENTOUS FLOWER*, maintain the middle domain and subsequently induces outgrowth of leaf lamina (Nakata et al., 2012). However, the precise mechanisms of lamina outgrowth along the medio-lateral axis are not fully understood.

These studies suggest that the mechanism underlying establishment of ad–ab polarity in leaves is both mutually antagonistic and robust as a result of the presence of redundant networks. However, mutants of *phb-1d*, *phv-1d*, *as1*, and *as2* show drastic phenotypes, such as radially symmetrical, trumpet-shaped, or peltate leaves (Waites and Hudson, 1995; McConnell and Barton, 1998; Xu et al., 2003). This suggests that modification and/or co-option of GRN for the ad–ab patterning can be associated with variations in leaf morphology. Moreover, understanding the GRN raises a general issue of whether it is associated with acquisition of flat structures or leaf-like organs, which remains an important question in plant morphological diversity.

## MODIFICATION OF GRN FOR LEAF DEVELOPMENT: UNIFACIAL LEAVES

**FIGURE 1:**
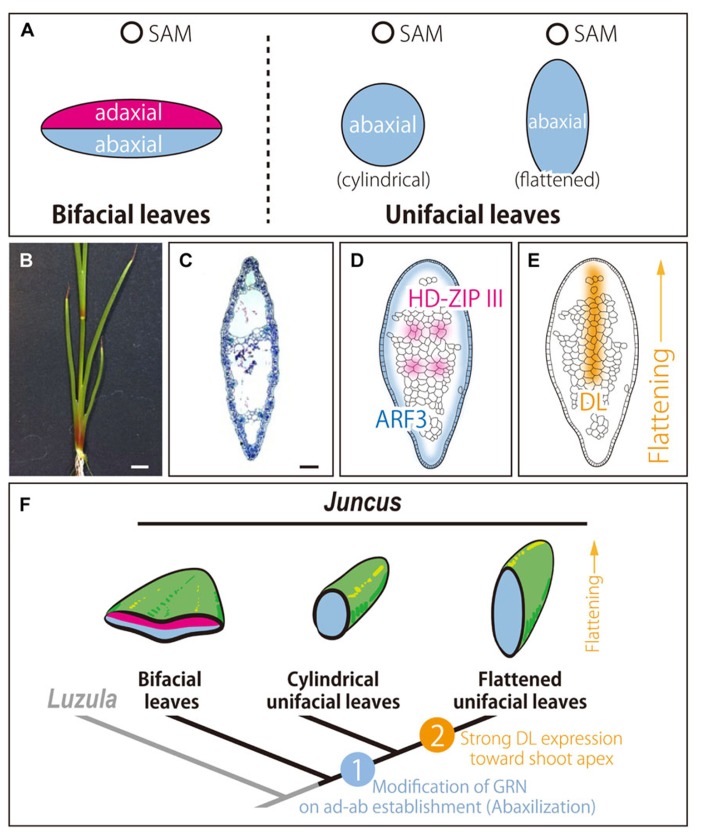
** Unifacial leaves in the genus *Juncus*.** **(A)** Schematic diagrams of leaf polarities in bifacial and unifacial leaves. **(B)** Seedling. **(C)** Transverse section of the leaf blade. **(D,E)** Expression patterns of orthologs in leaf primordia. *HD-ZIP III* and *ARF3* **(D)** and *DL* ortholog **(E)**. **(F)** Model of acquisition and blade flattening of unifacial leaves in the genus *Juncus*. **(B–E)** *J.* *prismatocarpus.* In **(C–F)**, top of the image is the adaxial side. Bars = 1 cm in **(B)** and 200 μm in **(C)**.

Unlike bifacial leaves common in angiosperms, unifacial leaves are characterized by an abaxialized leaf blade (Kaplan, 1975; **Figure 1A**). Interestingly, unifacial leaves have evolved repeatedly in monocots (Rudall and Buzgo, 2002; Yamaguchi and Tsukaya, 2010). However, the developmental and evolutionary processes of unifacial leaves remain largely uncharacterized, although several morphological studies have been performed. Many blades of unifacial leaves are flat despite being abaxialized, while establishment of ad–ab polarity is indispensable for lateral outgrowth of bifacial leaf blades (Waites and Hudson, 1995). This indicates that the flat form of leaves evolved independently in bifacial and unifacial leaves, and flat leaf blades in unifacial leaves may be regulated by mechanisms that differ from those of bifacial leaves (Yamaguchi and Tsukaya, 2010). Thus, unifacial leaves can be used as a model to investigate the evolution of leaf morphology, focusing on modification of ad–ab polarity (Kaplan, 1975; Rudall and Buzgo, 2002; Yamaguchi and Tsukaya, 2010).

Recently, Yamaguchi et al. (2010) explored the molecular mechanisms underlying the development and evolution of unifacial leaves in the genus *Juncus*. *Juncus prismatocarpus* has flat unifacial leaves (**Figures 1B,C**). In addition to morphological analysis, expression analysis was performed to confirm ad–ab polarity of leaf blades of *J. prismatocarpus*. As a result, a *HD-ZIP III* homolog was expressed in the presumptive region of xylem in procambial strands, whereas an *ARF3* homolog was expressed throughout the entire outer region of the leaf blade (**Figure 1D**). These results demonstrated that the leaf blade of unifacial leaves was abaxialized, as suggested by morphological observations. Additionally, the results suggested that modification of the GRN involved in establishment of ad–ab polarity in leaf may have resulted in abaxialized leaves in the genus *Juncus*. To our knowledge, this was the first case in which abaxialized leaves were confirmed by gene expressions of both adaxial and abaxial marker genes, excluding mutants of model species.

Moreover, the study using *Juncus* identified an alternative mechanism of leaf blade flattening without establishing ad–ab polarity by analyzing hybrids of a pair of closely related species; namely, *J. prismatocarpus* (with flattened unifacial leaves) and *J. wallichianus* (with cylindrical unifacial leaves). The results demonstrated that an ortholog of *DROOPING LEAF* (*DL*), a member of the *CRABS* *CLAW*/*DL* subfamily of *YABBY* genes, promotes flattening of blades of unifacial leaves in the genus *Juncus* (Yamaguchi et al., 2010; **Figure 1E**). The *DL* ortholog was strongly expressed in flattened unifacial leaves of *J. prismatocarpus*, whereas it was weakly expressed in cylindrical unifacial leaves of *J. wallichianus*. Genetic analysis using interspecific hybrids between the two species revealed that the *DL* locus from *J. prismatocarpus* flattens the unifacial leaf blade (Yamaguchi et al., 2010). An important function of *YABBY* genes is thought to be the promotion of directional cell proliferation in the lamina (Nakayama et al., 2010; Yamaguchi et al., 2012), in addition to the induction of leaf lamina-specific genetic programs and shut-down SAM programs (Sarojam et al., 2010). *DL* in *Oryza sativa* thickens the midrib by promoting cell proliferation toward the shoot apex (Yamaguchi et al., 2004). Similarly, the *DL* ortholog in unifacial leaves flattens the abaxialized leaf blades by promoting cell proliferation toward the shoot apex. This flattening by the *DL* ortholog may allow the leaf blades to stand straight to make the efficiency of light capture to be better.

These results suggested that modification of the GRN involved in the establishment of ad–ab polarity may have resulted in the evolution of unifacial leaves, and co-option of the GRN involved in directional cell proliferation may have resulted in the evolution of flattened unifacial leaves (Yamaguchi et al., 2010; **Figure 1F**).

## CO-OPTION OF GRN FOR LEAF DEVELOPMENT: CLADODES

**FIGURE 2:**
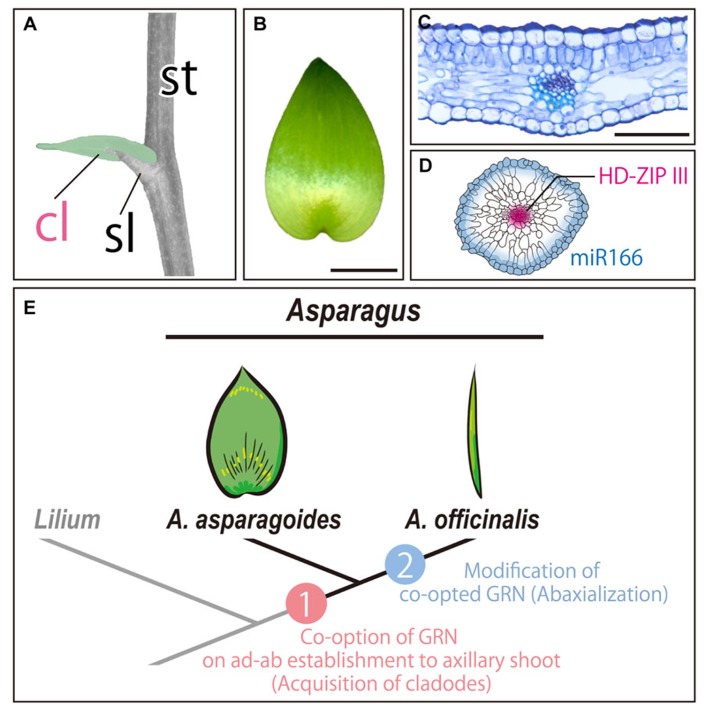
** Cladodes in the genus *Asparagus*.** **(A)** Generating the cladode position. A cladode is indicated by a false-color (green). **(B)** Gross morphology of a leaf-like cladode. **(C)** Transverse section of a leaf-like cladode. **(D)** Expression pattern of *HD-ZIP III* and miR166 in a rod-like (cylindrical) cladode. The image is a transverse section of a cladode. **(E)** Model of acquisition and morphological diversification of cladodes in the genus *Asparagus*. **(A–C)** *A. asparagoides*; **(D)** *A. officinalis*. cl, cladode; sl, scale leaf; st, stem. In **(C–D)**, the top of the image is the adaxial side. Bars = 1 cm in **(B)** and 1 mm in **(C)**.

Foliage leaves in the genus *Asparagus* are reduced in size. Instead, *Asparagus* has unusual organs called cladodes in the axils of scale leaves (**Figure 2A**). Interestingly, the morphology of cladodes is leaf-like, although it develops in an axil where a lateral shoot generally arises (**Figures 2A,B**). Therefore, cladodes have received much attention from the morphological and evolutionary standpoints. However, the uniqueness of cladodes makes it difficult to understand their origin. Arber (1924) concluded that cladodes are prophylls of abortive lateral shoots in the axils based on their leaf-like morphology and other anatomical features. Meanwhile, Cooney-Sovetts and Sattler (1986) and Kubitzki and Rudall (1998) concluded that cladodes are modified lateral branches based on their axillary position. Additionally, it is known that the morphology of cladodes in the genus is diverse (Kubitzki and Rudall, 1998; Fukuda et al., 2005). Recent molecular phylogenetic studies indicated that cladodes have evolved from a leaf-like (flattened) to a rod-like (cylindrical) form (Fukuda et al., 2005; Kubota et al., 2012). Currently, both the origin of and mechanisms underlying the diversification of cladodes remain unknown.

Nakayama et al. (2012a,b) investigated the molecular mechanisms underlying the acquisition and morphological divergence of cladodes in the genus *Asparagus*. Anatomical analysis demonstrated that leaf-like cladodes of *A. asparagoides* have ad–ab polarity (**Figure 2C**), and cell proliferation is concentrated in the proximal region. This suggested that the anatomy and development of cladodes are similar to those of leaves. However, the positions of the xylem and the phloem were inverted in cladodes compared to foliage leaves, suggesting that the cladodes differ from leaves (Nakayama et al., 2012a).

To further characterize the developmental processes of cladodes, expression analyses of orthologs of gene involved in leaf and/or shoot development were performed. The results showed that an ortholog of the *KNOX1* gene was expressed in the cladode primordia. However, its expression was limited to the peripheral region of primordia and ultimately decreased. Indeed, an ortholog of *AS1* was also expressed in the cladode primordia. Moreover, orthologous genes involved in the establishment of ad–ab polarity, such as *PHB*, *REV*, and miR166, were expressed in a leaf-like manner (Nakayama et al., 2012a). Thus, cladodes are modified axillary shoots that have evolved by co-option of the GRN and establish ad–ab polarity in the axillary shoots, as well as confer the leaf-like planar form (Nakayama et al., 2012a,b).

This was supported by investigations of *A. officinalis*, which has cylindrical cladodes (Nakayama et al., 2012a). Anatomical features such as the inner morphology and distribution of stomata on the epidermis indicated that cladodes of *A. officinalis* are abaxialized. In agreement with these results, a *PHB* ortholog was expressed in the presumptive region of vascular bundles, while miR166 was expressed throughout the outermost region of cladode primordia (Nakayama et al., 2012a; **Figure 2D**). Thus, the cylindrical form of the cladodes in *A. officinalis* can be attributed to the alteration of expression patterns of genes involved in the establishment of ad–ab polarity (Nakayama et al., 2012a). Molecular phylogenetic studies have suggested that the genus *Asparagus* has undergone rapid radiation in arid regions (Fukuda et al., 2005). The reduced surface area is thought to limit evapotranspiration and reduce water loss. Therefore, the morphological alteration from leaf-like to cylindrical forms likely contributed to the adaptation to arid and semiarid regions.

Overall, it was proposed that cladodes are modified axillary shoots, which have evolved by co-opting the GRN of leaf development. Subsequent alteration of the co-opted GRN has led to the cylindrical form of cladodes in the genus *Asparagus* (Nakayama et al., 2012a, b; **Figure 2E**). Therefore, the GRN of leaf development is likely required for the acquisition of leaf-like organs in the case of cladodes in the genus *Asparagus*.

## MODIFICATION AND CO-OPTION OF THE LEAF GRN IN PLANT MORPHOLOGICAL DIVERSITY

In unifacial leaves and cladodes, modifications and co-option of existing GRNs have led to the flat form of cylindrical leaves and lateral shoots. However, the flattening processes differ between the two cases. In *Asparagus* cladodes, the GRN involved in leaf development is thought to be co-opted. Therefore, it seems likely that the regulation of flattening and subsequent lamina outgrowth in cladodes is similar to that in leaves: ad–ab polarity dependent lamina outgrowth. This was supported by analysis of cylindrical cladodes in *A. officinalis* (Nakayama et al., 2012a).

On the other hand, in unifacial leaves of *Juncus*, flattening and subsequent lamina outgrowth are regulated by a distinct mechanism. For flattening, the *DL* ortholog promotes cell proliferation of abaxialized leaf primordia toward the SAM. Therefore, unlike bifacial leaves, flattening of primordia of unifacial leaves is independent of leaf ad–ab polarity. Additionally, the *DL* and *PRS* expression patterns demonstrated that subsequent marginal outgrowth is also independent of ad–ab polarity and occurs along newly established central–marginal axes. Analysis of *radial leaf* mutants in *J. prismatocarpus* indicated that the rearrangement is induced by initial leaf flattening and is important to marginal outgrowth of unifacial leaves in *Juncus* (Yamaguchi et al., 2010). These suggested the existence of multiple mechanisms of flattening originally cylindrical structures.

Recently, Katayama et al. (2010) reported that flattened roots in Podostemaceae, which is a family of aquatic angiosperms, have evolved by co-option of genes involved in shoot development. Therefore, the mechanism of root flattening also should be explored for further understanding of acquisition processes of planar forms. In nature, many plants show flattened structures such as phyllodes, winged stems, and pterocaul stems. Additionally, many of these structures, unifacial leaves, and cladodes have evolved repeatedly during plant evolution. Therefore, studies of *Juncus* and *Asparagus* will increase our understanding of acquisition processes of these flattened structures.

Knowledge gained from evo-devo studies of *Juncus* unifacial leaves and *Asparagus* cladodes has an interesting similarity: modifications of GRNs have led to abaxialization of each organ. Such abaxialization has been observed in diversified plant forms (e.g., Kim et al., 2003; Toriba et al., 2010). Therefore, abaxialization may be a common way in which novel structures and morphology arise, regardless of organ type. As opposed to abaxialization, adaxialization has not been reported to result in morphological diversification, although it leads to cylindrical structures (McConnell et al., 2001). One possible reason is the original identity of the leaf primordia. It is known that establishment of ad–ab polarity in leaf primordia requires an adaxializing signal from the SAM, which may be mediated by GABA shunt metabolites (Sussex, 1951; Toyokura et al., 2011). Microsurgical experiments that separate a primordium from a meristem tip generate an abaxialized leaf, indicating that the original identity of leaf primordia is abaxial. Thus, it may be difficult to convert the identity of the whole leaf primordia into adaxial identity. Alternatively, some sort of morphological or physiological differences between adaxial and abaxial sides may be subjected to evolutionary constraints. In fact, it is known that the adaxialization was easily caused by a simple mutation in miR165/166 target sites of *HD-ZIP* genes (McConnell et al., 2001; Rhoades et al., 2002). Nevertheless, the adaxialized organs or forms have not been seen in nature. Therefore, the latter hypothesis may be worth considering. In any case, further analyses will reveal whether abaxialization is a general trend during evolution of plants and constraints of adaxialization and abaxialization.

In this review, we focused on the relation between morphological diversification and alteration of GRNs for leaf development taking *Juncus* and *Asparagus* as examples. These instances indicate that modification and co-option of existing GRNs are an effective way to give rise to novel forms and organs. Additionally, these instances shed light on the importance of abaxialization in morphological diversity. However, many questions in alteration processes of GRNs remain unanswered. One is how specific part of newly co-opted GRN is modified without any perturbation of other developmental processes. The other is why specific GRNs are co-opted repeatedly in various contexts of development during evolution. In addition to revealing function of individual genes, elucidation of these questions about evolution of GRN will allow us to better understand how novel forms or organs arise in nature.

## Conflict of Interest Statement

The authors declare that the research was conducted in the absence of any commercial or financial relationships that could be construed as a potential conflict of interest.
